# Menopausal Symptoms and Utian Quality of Life Scale Following a Breast Cancer Diagnosis and Its Impact on Endocrine Adherence

**DOI:** 10.7759/cureus.52777

**Published:** 2024-01-23

**Authors:** Norah P Scally, Lara Armstrong, Daryl Blades, Eimer McGeown, Helen Mathers

**Affiliations:** 1 Department of Breast Surgery, Southern Health and Social Care Trust, Portadown, GBR; 2 Department of General Surgery, Southern Health and Social Care Trust, Portadown, GBR

**Keywords:** cancer survivorship, hormone receptor-positive breast cancer treatment, quality of life in breast cancer patient, menopause rating scale, breast cancer outcomes

## Abstract

Introduction

Standard treatment for oestrogen-positive breast cancers involves a minimum of five years of adjuvant endocrine treatment with a significant improvement in survival. However, the side effects of endocrine treatments are often underestimated. We aimed to identify the frequency of side effects, adherence to treatment, and impact on the quality of life of breast cancer survivors.

Methods

All patients attending holistic needs assessment and health and wellbeing events with a clinical nurse specialist between March and October 2023 were given a menopause symptom proforma and Utian menopausal quality of life scale questionnaire.

Results

A total of 99/150 (66%) patients attending a health needs assessment with a clinical nurse specialist following a diagnosis of breast cancer returned forms.

The mean age of respondents was 56.7 years, with a mean 2.5-year duration since diagnosis. Thirty-seven percent of respondents were premenopausal at diagnosis, and 63% were postmenopausal. Five percent stopped treatment early due to menopausal symptoms, and 2.2% changed endocrine treatment. Overall, the mean menopausal quality of life score was -0.454 (p=0.0052). Within the premenopausal cohort, 84% reported hot flushes, 81% a low-sex drive, 73% night sweats, 89% memory problems, 89% fatigue, and 76% joint aches. This group scored -0.20 SD on the quality of life scale. The postmenopausal group reported a 71% incidence of hot flushes, 79% both poor sleep and joint pain, 60% breast pain, and 86% fatigue. They demonstrated a mean of -0.58 SD on the quality of life scale. The failure to adhere to endocrine treatment was reported by 6% of respondents, who cited side effects as the reason for non-compliance.

Conclusion

In conclusion, there is a significant increase in menopausal symptoms following treatment for breast cancer, which is negatively impacting well-being, quality of life, and endocrine adherence.

## Introduction

Breast cancer remains the most common female cancer and the leading cause of cancer-related deaths in women [1]. Menopause has become a controversial topic of societal debate, with a greater general awareness of symptoms. Women are expected to experience menopausal symptoms for approximately one-third of their lifetimes [2]. Up to 70% of breast cancer diagnoses demonstrate oestrogen receptor (ER) positivity, targeted with standard adjuvant endocrine treatment. There are estimated increased survival rates for oestrogen-positive cancer at 90%-92.5% over a four-year survival timeframe [3]. However, women are living long after their breast cancer diagnosis in a medically-induced premature oestrogen deficit.

Menopausal symptoms and side effects of anti-oestrogen treatment have a direct impact on quality of life and adherence to medication and can potentially negatively impact survival [4]. An important aim of cancer care should be a return to pre-cancer quality of life encompassing emotional, physical, and social health.

The aim of this study was to determine the most common side effects of treatment in the first three years post diagnosis and to determine the impact of those side effects on occupational and emotional quality of life, as well as health and sexual functioning.

## Materials and methods

All patients diagnosed with primary breast cancer in Southern Health and Social Care Trust, Portadown, GBR, a single healthcare trust were invited to a face-to-face holistic needs assessment (HNA) with a clinical nurse specialist and also to a health and wellbeing group event. Patients were identified from a unit database of diagnoses, and all patients undergoing primary treatment in the past three years were eligible for an invitation. All patients attending the two health and wellbeing events during seven months between March and October 2023 and all HNAs in the same period were included. All complete questionnaires returned up to January 2024 were included.

At each event, patients were given a questionnaire of 30 of the most common menopausal symptoms as described by the British Menopause Society and a Utian menopausal-validated quality of life questionnaire and asked to return anonymously by post (Appendix A). The Utian quality of life tool is a validated tool for assessing the quality of life peri- and postmenopausally [5] and was utilized and interpreted using the recommended methodology for analysis and scoring.

Patients were asked to indicate the presence of symptoms before and after cancer diagnosis, as well as the type of endocrine treatment, adjuvant chemotherapy, and whether they were still complying with treatment.

Data were collated on a Microsoft Excel (Microsoft Corp., Redmond, WA) database. The p-values were calculated using GraphPad Prism software (GraphPad Software, La Jolla, CA), Fisher's exact t-test, and independent t-test calculators.

Exclusions included a diagnosis of metastatic disease, incomplete returned questionnaires, and patients undergoing primary endocrine treatment.

## Results

A total of 100 patients completed questionnaires out of a potential 53 patients attending health and wellbeing events and 97 patients having a one-to-one HNA (66% return) between March and October 2023. One incomplete questionnaire was excluded as no demographic data were filled out.

Within the total group, the average age was 56.7 years (range: 35-81 years), with 37/99 premenopausal and 62/99 postmenopausal at the time of diagnosis.

The two subsets were independently analysed. The premenopausal group had a mean of 2.6 years from diagnosis and the postmenopausal group 2.4 years. The average age was 50.2 years in the premenopausal group, compared to 63.3 years in the postmenopausal group (Table 1).

**Table 1:** Demographics of patient groups

	Premenopausal	Postmenopausal	Total
Total number	37	62	99
Mean age	50.2 years	63.3 years	56.7 years*
Mean time from diagnosis	2.6 years	2.4 years	2.5 years
Adjuvant chemotherapy	45.6%	27%	34%
Adjuvant hormones			
No hormones	10.8%	11.3%	11.1%
Tamoxifen	75.7%	11.3%	35.4%
Aromatase inhibitor	0	77.4%	48.5%
Aromatase inhibitor + ovarian suppression	13.5%	0	5%
Percentage who stopped treatment	6.1% (2/33)	5.9% (3/51)	6%

In the premenopausal cohort, 4/37 (10.8%) were ER-negative at diagnosis, 28/37 (75.6%) received tamoxifen, and 5/37 (13.5%) received an aromatase inhibitor with ovarian suppression; 45.6% (n=17) received adjuvant chemotherapy. Thirty-two percent of this group considered themselves perimenopausal, and 68% considered themselves premenopausal.

The questionnaire asked about the pre-and post-diagnosis occurrence of 30 frequent menopausal symptoms, as described by the British Menopause Society. Of these, there was a statistically significant increase in hot flushes (11%, n=4) pre-treatment and 84% (n=31) post-treatment, night sweats, irritability, low sex drive, fatigue, memory issues, joint aches, weight gain, and breast pain, with a p-value <0.05 (Table 2).

**Table 2:** Incidence of menopausal symptoms post treatment N: total number of patients describing symptoms; %: percentage of cases describing symptoms in each subgroup; p: p-value is calculated using Fisher's exact test to determine the difference from pre-treatment incidence in each group.

Symptom	Premenopausal			Postmenopausal			Total as %
	N	%	p	N		%	
Hot flushes	31	84%	<0.001	23		37%	54%
Cold flashes	8	22%		15		24%	23%
Night sweats	27	73%	<0.001	40		65%	67%
Heart palpitations	10	27%		24		39%	32%
Irritability	24	65%	0.0009	28		45%	52%
Mood swings	24	65%		32		52%	59%
Trouble sleeping	31	84%		49		79%	80%
Irregular periods	6	16%		1		2%	7%
Low sex drive	30	81%		32		52%	62%
Vaginal dryness	24	65%		33		53%	57%
Fatigue	33	89%		50		81%	83%
Anxiety	24	65%		39		63%	63%
Depression	14	27%		27		44%	41%
Poor concentration	30	81%		33		53%	63%
Faulty memory	33	89%		32		53%	65%
Incontinence	8	22%		15		24%	23%
Achy joints/muscles	28	76%		44		79%	73%
Headaches	17	46%		18		29%	35%
Bloating	18	49%		17		27%	35%
Weight gain	25	76%		32		55%	57%
Hair loss/thinning	17	46%		24		39%	41%
Facial hair	10	27%		21		34%	31%
Dizziness	9	24%		19	31%		28%
Vertigo	8	22%		9		15%	17%
Changed body odour	5	14%		14		23%	19%
Tingling extremities	14	27%		30	48%		44%
Osteoporosis	6	16%		19	31%		25%
Weakened fingernails	14	27%		27	44%		41%
Tinnitus	12	32%		7	11%		19%
Breast pain	19	51%		37	60%		56%

Within the postmenopausal group 1, n=7 were ER-negative, with 77.4%, n=48 on an aromatase inhibitor, and 11.3% (n=7) on tamoxifen. There was a significant increase in hot flushes, night sweats, irritability, trouble sleeping, low sex drive, memory problems, joint aches, weight gain, and breast pain (Table 2).

Within the premenopausal group, 6.1% (n=2) had stopped endocrine treatment, which they attributed to the side effects of medication. A similar 5.9% (n=3) stopped taking endocrine treatment at 2.4 years in the postmenopausal group.

Analysis of the Utian menopausal quality of life scale in the postmenopausal group demonstrated a significantly lower score with a total mean score of 66.6, or -0.58 standard deviation (SD) from the test mean (p=0.0059). The premenopausal group, however, had a mean total score of 71.6, or -0.2 SD from the mean (p=0.4233). The overall mean total group score was -0.454 SD, which was significantly different from the mean control test population (p =0.0052). Within the four subsets of menopausal quality of life, i.e., occupational, health, emotional, and sexual, there was only a statistically significant difference in quality of life in the occupational subset with a p<0.0001. Within the health quality of life, the p=0.1547, the emotional p=0.29, and within the sexual menopausal quality of life, there was no significant difference reported (p = 0.8876). The scores are presented in Table 3.

**Table 3:** The Utian menopausal quality of life score as SD from mean Values are represented as standard deviations (SD) from the control group mean. The p-values represent the t-test comparison to the control group scale mean value.

Utian menopausal quality of life score as SD from mean	Premenopausal		Postmenopausal		Total score	
Total score	-0.20	p=0.4233	-0.58	p=0.0059	-0.454	p=0.0052
Occupational score	-0.56	p=0.07	-1.14	p<0.0001	-0.936	p<0.0001
Health score	+0.13		+0.22		0.1804	p=0.1547
Emotional score	+0.06		-0.26		-0.162	p=0.2929
Sexual score	+0.22		-0.13		-0.015	p=0.8876

## Discussion

This study demonstrates that both pre-and postmenopausal women following a diagnosis of breast cancer experience a statistically significant increase in reported menopausal symptoms. An increase in all 30 menopausal symptoms was demonstrated in both premenopausal and postmenopausal cohorts. However, some symptoms were more prevalent. There was a marked increase in hot flushes, with 84% of premenopausal patients and 71% of postmenopausal women describing this as a side effect of endocrine treatment (an increase of 34% from baseline pretreatment). The most frequent complaint in postmenopausal patients was trouble sleeping (34% to 79% incidence after treatment) and joint aches (24% to 79% incidence). The overall incidence of any of the 30 most frequent symptoms was 46%; this is higher than previously described by Hong et al. [6], who described a mean incidence of hot flushes of 30% in aromatase inhibitor users alone.

Within the premenopausal group, the most frequently described symptoms were fatigue and poor memory at 89% and joint aches at 76%. Despite the higher reported incidence of symptoms in premenopausal patients, their overall menopausal quality of life score was not statistically significantly lower than the Utian menopausal quality of life mean score of -0.20 (p=0.4233). In particular, the premenopausal cohort scored higher than average in health, emotional, and sexual scores (Table 3), although this was not significant. The Utian menopausal quality of life scale is designed to assess a perception of well-being rather than purely menopausal symptoms [5]. As such, despite the higher reported symptoms, the overall quality of life appears to be unaffected in this cohort. Perhaps the lower numbers of premenopausal women in the group underpowered the results of this cohort, and further studies on the quality of life of breast cancer survivors, particularly in the premenopausal group, would be useful.

Within the overall group, the mean time from diagnosis was 2.5 years, with a 6% failure to adhere to endocrine treatment. In all cases, this was attributed to the side effects of medication. Previous studies have suggested a 25% failure to adhere by five years [6] with a significant effect on survival. Although this is a lower failure to comply, it is still a concerning figure at a time when breast cancer recurrence rates are known to peak. Freedman et al. described a higher incidence of noncompliance in a younger population [7], but with 6.1% and 5.9% failure rates in this study, we found no difference between the groups.

Within the postmenopausal group, there was a significant increase in menopausal symptoms and a significant reduction in quality of life. However, the overall incidence of symptoms appeared lower in the postmenopausal group compared to the premenopausal patients. Hong et al. described a 17% incidence of fatigue and arthralgia in this population [6], in contrast to the 79% and 81% described in our cohort. However, in this cohort, there was a statistically significant reduction in perceived quality of life and well-being (mean=-0.58, p=0.0059). A proposed hypothesis for fewer symptoms but poorer quality of life may be that the return of menopausal symptoms for a second time has a greater effect on psychological well-being. Previous studies have certainly shown a reduced adherence to endocrine treatment with increasing age in a postmenopausal population [6, 8].

The premenopausal cohort was also more likely to have completed adjuvant chemotherapy (45.9% vs. 27% in the postmenopausal group). This is likely to have contributed to the higher incidence of fatigue (81%), as well as other post-treatment side effects. Di Nardo et al. described a 50% incidence of fatigue at five years with a significant reduction in quality of life following chemotherapy, in contrast to the results of this study [9]. This could be attributed to the reduction in side effects with time. However, analysis of this chemotherapy subgroup showed no significant difference in the quality of life wellbeing score (mean chemotherapy group: -0.64, mean non-chemotherapy group: -0.36, p=0.413) compared to those who didn’t receive chemotherapy.

Overall, in the combined analysis, however, there was a statistically significant reduction in menopausal quality of life scores. As this scale was validated on women who were peri- and postmenopausal [5] and this study group had an average age of 56.7 years, the groups are comparable. This group of patients would expect to be postmenopausal, with an average age of menopause in the United Kingdom being 51 years [10]. Of particular note, there was a significant impact on occupational well-being in both cohorts, which drove the overall score to be significant. Schmidt et al. described a 57% return to work at one year in premenopausal women following a breast cancer diagnosis, with a significant impact on quality of life and a return to work continuing beyond five years. This study strongly correlates a reduction in occupational quality of life as a result of menopausal symptoms in keeping with both Schmidt et al. and Bijker et al. [11, 12].

Within the subsets of emotional, sexual, and health, there was not a statistically significant change from the expected mean well-being score. Within the premenopausal group, 81% described a low sex drive and 65% vaginal dryness, compared to 62% and 57%, respectively, in the postmenopausal group. Dinapoli et al. described a 76% incidence of sexual dysfunction following a breast cancer diagnosis in all age groups [13], in keeping with the levels described in our cohort. These results give weight to the need for advice and help with sexual functioning for both pre-and postmenopausal women. However, given this high incidence of side effects on sexual functioning, there was no impact on sexual quality of life (mean -0.015 SD, p=0.8876)

Antunozzo et al. described a significant reduction in fatigue duration with the provision of information leaflets and phone-based nurse monitoring assessments akin to the HNA provided to these patients [14]. While Ng et al. described the positive effects of holistic needs assessments and their role in addressing the complex social, psychological, and physical needs of breast cancer survivors [15], the reduction in quality of life, coupled with the fact that the questionnaires were completed after attendance at an HNA with a clinical nurse specialist, where help and signposting to support had already been provided, is of particular note. It would be expected that their attendance at health and wellbeing events and HNA might bias the results in a positive manner, which further adds weight to the significance of the negatively significant results obtained.

Further limitations of this study include the small subsets with only 37 and 62 patients in the premenopausal and postmenopausal groups, respectively, which can be explained by the poorer turnout at the health and wellbeing events than would normally be expected given that this was their first return post-COVID. However, concordance with the results and previously described incidence of menopause symptoms [16] adds to their weight in identifying the correlation between menopausal symptoms, quality of life, and endocrine adherence.

## Conclusions

This study identifies the correlation between menopausal symptoms, quality of life, and adherence to endocrine treatment in the early years after breast cancer diagnosis. We particularly note the negative effects of treatment on an older postmenopausal population.

Given that we recognise the significant reduction in survival as a result of noncompliance with endocrine treatment, this study adds weight to the need for a better understanding of and support for the physical, social, sexual, emotional, and psychological effects of breast cancer treatment and the impact on wellbeing in breast cancer survivors.

We draw particular attention to the multiple menopausal symptoms that have increased as a result of endocrine treatment and call for targeted support strategies to be incorporated into routine survivorship care.

## Appendices

Appendix A

**Table 4:** Questionnaire used to gather patient responses.

Age	
Age at diagnosis	
Chemotherapy (yes /no)	
Menopausal status at diagnosis	
Current menopausal status	
Hormone treatment ( eg tamoxifen, Letrozole)	
Duration of hormone tablet	
Current hormone tablet (yes/no)	
Did you stop taking the tablet early? Is so Why	

Symptom	Pre-treatment	Current
Hot flushes		
Cold flashes		
Night sweats		
Heart palpitations		
Irritability		
Mood swings		
Trouble sleeping		
Irregular periods		
Low sex drive		
Vaginal dryness		
Fatigue		
Anxiety		
Depression		
Poor concentration		
Faulty memory		
Incontinence		
Achy joints/muscles		
Headaches		
Bloating		
Weight gain		
Hair loss/thinning		
Facial hair		
Dizziness		
Vertigo		
Changed body odour		
Tingling extremities		
Osteoporosis		
Weakened fingernails		
Tinnitus		
Breast pain		
